# Mechanistic insights into high-intensity ultrasound-induced debittering and umami-enhancing of Antarctic krill peptides during ultrasound-assisted flavourzyme hydrolysis: Integrating E-tongue, multi-spectroscopy, and label-free peptidomics^☆^

**DOI:** 10.1016/j.ultsonch.2026.108031

**Published:** 2026-08-28

**Authors:** Haoyu Liu, Jiaxin Chen, Ruoyu Xie, Jun Hu, Hongying Du, Xinyan Peng, Zhengshun Wen, Xiaolong Ji, Wenxuan Chen, Jin Zhang

**Affiliations:** a State Key Laboratory for Quality and Safety of Agro-products, Zhejiang Key Laboratory of Intelligent Food Logistic and Processing, Institute of Food Science, Zhejiang Academy of Agricultural Sciences, Hangzhou, Zhejiang 310021, PR China; b Department of Food Science and Engineering, College of Light Industry and Food Engineering, Nanjing Forestry University, Nanjing 210037, PR China; c College of Life Science, Yantai University, Yantai, Shandong 264005, PR China; d Xianghu Laboratory (Zhejiang Laboratory of Agriculture), Hangzhou, Zhejiang 311231, PR China; e College of Food and Bioengineering, Zhengzhou University of Light Industry, Zhengzhou, Henan 450001, PR China

**Keywords:** Antarctic krill, By-product, Peptide, Ultrasound-assisted enzymolysis, Bitterness, Umami, Peptidomics

## Abstract

The bitterness of protein hydrolysates negatively affects the utilization of protein-rich by-products from Antarctic krill after oil extraction. The debittering and umami-enhancing effects as well as the underlying mechanisms of high-intensity ultrasound on Antarctic krill peptides (AKPs) during ultrasound-assisted flavourzyme hydrolysis were unveiled by integrating E-tongue, multi-spectroscopic, and peptidomic approaches. Results revealed that appropriate high-intensity ultrasound allowed ordered conformations (α-helices and β-sheets) to convert into disordered conformations (mainly β-turns), promoting the unfolding of structural proteins (mainly myofibrillar proteins) and cleavage of exposed hydrophobic residues during enzymolysis, which improved the protein recovery and degree of hydrolysis (DH) of AKPs. These changes led to the small-molecular weight (MW) proportion (<3 kDa) increase, peptide length shortening, N-terminal/C-terminal hydrophobic residues reduction, hydrophobic/bitter residues (especially Phe, Ile, Leu, Met, Pro, and Val) content decline, and hydrophilic/umami residues (mainly Asp and Glu) amount augment of AKPs, contributing to the generation of fewer underlying bitter peptides and release of more potential umami peptides, which resulted in the bitterness-associated perceptions (bitterness, bitter aftertaste, and astringency) attenuation and umami-related tastes (umami and richness) enhancement. However, overhigh-power (>400 W) high-intensity ultrasound could disturb α-helices degradation along with exposure and cleavage of hydrophobic residues during enzymolysis, which would decrease the DH and protein recovery, leading to the bitterness enhancement and umami decline of AKPs through augmenting average MW, increasing hydrophobic/bitter residues proportion, and reducing hydrophilic/umami residues content. These findings provided insights into the high-intensity ultrasound-induced taste-improving mechanism of peptides and facilitated the high-value utilization of defatted Antarctic krill by-products.

## 1 Introduction

Antarctic krill (*Euphausia superba*), one of the most abundant species in the Southern Ocean, has an estimated total biomass of 300–500 million tons [1]. Antarctic krill oil, accounting for 15–20 % of the whole krill on a dry basis, is gaining increasing attention among worldwide consumers owing to its rich reserves of phospholipids, ω-3 polyunsaturated fatty acids, astaxanthin, and other nutrients [2], [3]. Consequently, Antarctic krill is primarily used to produce the krill oil in the food industry, whereas the by-products post-oil extraction are typically discarded or only processed to low-value commodities, such as animal feeds or feed attractants [1], [4]. These defatted by-products, containing 65–75 % proteins on a dry basis, are considered a sustainable marine protein resource due to their low cost, large production, well-balanced amino acid composition, high biological value, and good digestibility [1], [2]. More importantly, Antarctic krill protein has been widely reported as a good source of peptides with diverse bioactivities, such as metal ion chelation [5], xanthine oxidase inhibition [6], gut microbiota modulation [7], memory improvement [8], and immunoregulation [9]. Therefore, it is of great significance to develop these defatted high-protein by-products into functional foods or ingredients rich in peptides.

However, the high ratio of hydrophobic and bitter amino acid residues (especially aromatic residues) results in the undesirable bitterness inherent of Antarctic krill peptides (AKPs) or protein hydrolysates, which simultaneously masks their favorable umami imparted by abundant Glu and Asp residues, negatively affecting their sensory quality and limiting their utilizations in food or pharmaceutical industries [2], [5], [10]. Bitterness and its related tastes are a common disadvantage to be addressed for many protein hydrolysates or peptides. It can be influenced by various factors, including molecular size, average hydrophobicity, the proportion of bitter amino acid residues, the composition of N-terminal and C-terminal residues, and chain conformation [10], [11], [12]. In light of these contributors, several debittering strategies are currently under research and development, which can be categorized into physicochemical debittering (physical modification, Maillard reaction, bitterness masking, *etc.*) and biological debittering (enzymolytic optimization, plastein reaction, *etc.*) [10].

Flavourzyme, a complex protease isolated from *Aspergillus oryzae*, is increasingly used for the preparation of bitterness-reduced peptides because it has both endopeptidase and exopeptidase activities, which are conducive to cutting off the terminal hydrophobic residues of peptides [13]. However, as the complex conformations of substrate proteins prevent the contact between its exopeptidase and peptide chain terminus, flavourzyme is usually associated with a relatively lower degree of hydrolysis (DH) compared with many other commercial proteases, such as pepsin, neutrase, and alcalase, which would limit the debittering effect, protein recovery, and peptide quality [14], [15]. On the other hand, high-intensity ultrasound, as a high-efficiency, cost-effective, and eco-friendly physical processing method, has been widely used to assist the protein utilization from food by-products [16]. Unlike the low-intensity ultrasound (<1 W/cm^2^, 100 kHz-1 MHz) and its gently mechanical effects, high-intensity ultrasound (>1 W/cm^2^, 20-500 kHz) is capable of rapidly altering structural and functional properties of food macromolecules through its strong cavitation effects and high-energy density [16]. Plenty of previous studies have proved that high-intensity ultrasound could promote protein conformational alteration, increase hydrolysate yield, and enhance peptide DH during enzymolysis [17], [18], [19], which could compensate for the above shortcomings of flavourzyme hydrolysis. Therefore, the high-intensity ultrasound-assisted flavourzyme hydrolysis (HUF) can be a promising peptide-debittering approach that combines both physicochemical and biological strategies.

Nevertheless, the influences of HUF on taste attributes (especially bitterness and umami) of peptides were rarely investigated. Besides, the optimal HUF conditions (especially ultrasonic power) used for good tastes of peptides were also not confirmed. Furthermore, the underlying mechanisms associated with high-intensity ultrasound were still not fully understood. Hence, AKPs were prepared from Antarctic krill powders by HUF with different ultrasonic powers in the present work. The objective of this study was i) to investigate the influences of HUF powers on bitter and umami perceptions of AKPs through sensory evaluation, E-tongue analysis, and chemometrics; and ii) to unlock the high-intensity ultrasound-induced taste-improving mechanisms during this process by integrating multi-spectroscopic, label-free peptidomic, and multivariate statistical approaches. The information obtained from this research is of great significance to the high-value utilization of processing by-products and improvement of the whole economic value for the Antarctic krill industry.

## 2 Materials and methods

### 2.1 Materials

Antarctic krill (*Euphausia superba*) powders were sourced from Nutrition Biotechnology Co., Ltd. (Hangzhou, China). The flavourzyme (200,000 U/g) was bought from Pangbo Biological Engineering Co., Ltd. (Nanning, China). The bromophenol blue (BPB) solution (1 mg/mL, w/v) was purchased from Yuanye Bio-technology Co., Ltd. (Shanghai, China). The Antarctic krill powders, flavourzyme, sucrose, salt, citric acid, L-leucine, monosodium glutamate (MSG), CaCl_2_, and NaHCO_3_ were of food grade, whereas other reagents and chemicals used were of analytical grade.

### 2.2 Peptide preparation through high-intensity ultrasound-assisted flavourzyme hydrolysis (HUF)

Based on the methods of Su et al. [3], Yue et al. [7], and Huang et al. [19] with some modifications, Antarctic krill powders were defatted, ultrasound-assisted enzymolyzed, and defluorinated to obtain peptides. Firstly, the powders were mixed with ethanol (1:10, w/v) and stirred at 1000 rpm for 5 h (MYP11-2, Heying Instruments Co., Ltd., Shanghai, China) to defat. Then the mixture was vacuum-filtered and the collected precipitate was dried at 40 °C for 10 h in a DNG-9246A electric air-drying oven (Jinghong Laboratory Equipment Co., Ltd., Shanghai, China). Afterward, the defatted powders were dissolved in water (1:14, w/v), adjusted to pH=7.0, and added with flavourzyme (500 U/g, w/w). The HUF treatment was carried out using a SB25-12DTD ultrasonicator with a maximum power of 720 W (Scientz Biotechnology Co., Ltd., Ningbo, China) at the 40 kHz ultrasonic frequency, 0-700 W ultrasonic power, 50 °C enzymolytic temperature, and 4 h enzymolytic time.

Subsequently, the hydrolysates were heated in boiling water for 10 min to terminate the enzymolysis, followed by cooling to ambient temperature and centrifuged at 8000 rpm for 20 min (Avanti J-26XP, Beckmen Coulter, Fullerton, CA, USA). Then the collected supernatant was added with 1% CaCl_2_ (w/w), adjusted to pH=8.0, and stirred at 700 rpm and room temperature for 1 h, followed by another centrifugation at 8000 rpm for 20 min to remove fluorides. The supernatant (defluorinated hydrolysate) was collected and freeze-dried at -50 °C for 48 h (SCIENTZ-12N, Scientz Biotechnology Co., Ltd., Ningbo, China) to obtain Antarctic krill peptide (AKP) powders, which were stored at -80 °C until use.

### 2.3 Determination of protein recovery and degree of hydrolysis (DH)

The protein recovery rate and DH were determined as described by Li et al. [4] and Sun et al. [20] with minor modifications. Briefly, the total protein content of Antarctic krill powders and total protein nitrogen of defluorinated hydrolysates were determined by the Kjeldahl method following the procedure of ISO 1871-2009. The α-amino nitrogen of defluorinated hydrolysates was measured through the formol titration method, while the AKP content (solid content) of defluorinated hydrolysates was detected with the direct drying method following the procedure of ISO 1442-2023. The protein recovery and DH were calculated with the following equations, respectively:

(1) $$\text{Protein recovery}\;\left(\%\right)=\left(C_{\mathrm{AKP}}\times V\right)/\left(C_{\mathrm{TP}}\times m\right)\times 100\%$$

(2) $$\mathrm{DH}\left(\%\right)=C_{\mathrm{AN}}/C_{\mathrm{TPN}}\times 100\%$$

where *C_AKP_* was the AKP content of defluorinated hydrolysates, mg/mL; *V* was the volume of defluorinated hydrolysates, mL; *C_TP_* was the total protein content of Antarctic krill powders used, mg/g; *m* was the mass of Antarctic krill powders used, g; *C_AN_* was the α-amino nitrogen content of defluorinated hydrolysates, M; *C_TPN_* was the total protein nitrogen of defluorinated hydrolysates, M.

### 2.4 Sensory evaluation of taste

Based on the procedure of Wang et al. [21] and Mei et al. [22] with minor modifications, the sensory quality of taste was evaluated by an experienced sensory panel consisting of eight members (four males and four females, aged from 22 to 35) without smoking history or taste/odor disorders. All panelists were trained according to the procedure of a Chinese national sensory evaluation specification for aquatic foods (GB/T 37062-2018) by an expert from Zhejiang Academy of Agricultural Sciences. Specifically, a series of taste reference solutions were thoroughly scored by all panelists before sensory evaluation, including 0.6% sucrose for sweetness, 0.08% citric acid for sourness, 0.35% MSG for umami, 0.35% salt for saltiness, and 0.5% L-leucine for bitterness. The taste session was executed in a quiet room with natural light and fluorescent lamps. A 10-point scale (10=extremely strong and 0=none) was used to describe the intensity of each taste attribute of AKP solutions (redissolved to 4 mg/mL with deionized water), and the taste intensities of all reference solutions were given a score of 5. To avoid intercommunications and interferences, each panelist was spaced between each other, and a gargle with warm clean water was required between two tastings of different groups.

### 2.5 Electronic tongue (E-tongue) analysis

The taste was also objectively analyzed using a SA402B E-tongue (INSENT Co., Ltd., Kyoto, Japan) based on the method of Wu et al. [23] with minor modifications. Specifically, five taste sensors were equipped to thoroughly measure basic taste attributes, including CA0 for sourness (tasteless point: -13), C00 for bitterness and bitter aftertaste (tasteless point: 0), AAE for umami and richness (umami aftertaste) (tasteless point: 0), AE1 for astringency and astringent aftertaste (tasteless point: 0), and CT0 for saltness (tasteless point: -6). Meanwhile, two reference electrodes were also equipped with 0.3 mM tartaric acid and 30 mM KCl as the reference solution, respectively. The AKP powders were adjusted to 4 mg/mL (w/v) solutions using deionized water for the measurement, and taste sensors were activated before the measurement to ensure accuracy. Each sample was detected for four cycles, and results of the first cycle were discarded while data from the other three cycles were retained for analysis.

### 2.6 Multi-spectroscopic characterization

#### 2.6.1 Ultraviolet-visible (UV-vis) spectroscopy

The UV-vis spectrum was obtained through a UV-2600i spectrophotometer (SHIMADZU Co., Ltd., Kyoto, Japan) as described by Huo et al. [18] with slight modifications. Briefly, defatted Antarctic krill or AKP powders were redissolved to 1 mg/mL solutions with deionized water and transferred to a cuvette for the analysis within 200-800 nm at the resolution of 1 nm. Prior to the measurement, the deionized water was used for the baseline calibration to eliminate the solvent interference.

#### 2.6.2 Intrinsic fluorescence spectroscopy

The intrinsic fluorescence intensity was assessed using an F-7000 spectrofluorometer (Hitachi Co., Ltd., Tokyo, Japan) based on the method of Li et al. [4] with some modifications. Briefly, 0.2 mg/mL defatted Antarctic krill or AKP solution prepared with 10 mM PBS (pH=7.0) was excited at 290 nm, and the emission spectrum was collected within the wavelength range of 310-460 nm. Besides, the spectral scan speed was set as 1200 nm/min with a 5 nm slit for both excitation and emission spectrum.

#### 2.6.3 Fourier transform-infrared (FT-IR) spectroscopy

Based on the method of Zhang et al. [24] with slight modifications, the chemical structure was analyzed through a VERTEX70 FT-IR spectrometer (Bruker AXS, Karlsruhe, Germany). The defatted Antarctic krill or AKP powders were mixed with KBr and compressed to semitransparent pellets for analysis. The FT-IR analysis was conducted at the following parameters: sample/KBr ratio, 1:100 (w/w); wavenumber range, 400-4000 cm^-1^; scanning frequency, 32; and resolution, 4 cm^-1^. The spectra data were normalized, Gaussian deconvoluted, and then second derivative fitted using the OMNIC software V8.0 (Thermo Fisher Scientific, Madison, WI, USA) and the PeakFit software V4.12 (SeaSolve Software Inc., Framingham, MA, USA) to calculate the relative contents of different secondary structures (α-helix, β-sheet, β-turn, and random coil) from amide I bands (1600-1700 cm^-1^), which were expressed as their percentages of areas under corresponding sub-bands.

#### 2.6.4 Circular dichroism (CD) spectroscopy

The CD spectroscopic detection was carried out with a J1500 spectrometer (Japan Spectroscopic Co., Ltd., Tokyo, Japan) following the procedure of Fan et al. [5] with some modifications. Briefly, the AKP powders were prepared as 0.5 mg/mL solutions using 2.5 mM PBS (pH=7.0) and transferred to a 1 mm cuvette, followed by the measurement within 190-400 nm at a scan speed of 100 nm/min. Specially, the above-mentioned PBS was used for the baseline calibration to eliminate the solvent interference before the measurement. The relative contents of secondary structures were approximately estimated through the CDNN V2.1 program by a neural network-based algorithm.

#### 2.6.5 X-ray diffraction (XRD) spectroscopy

According to the method of Chen et al. [25] with slight modifications, the XRD spectroscopic measurement was performed using an Ultima IV diffractometer (Rigaku Holdings Co., Ltd., Tokyo, Japan) at the following conditions: illuminant, Cu-Kα ray; tube voltage, 40 kV; tube current, 40 mA; scanning speed, 2^o^/min; diffraction angle 2θ, 5-60^o^.

### 2.7 Surface hydrophobicity assay

The surface hydrophobicity was assayed as the BPB-binding amount using the method of Yang et al. [26] with minor modifications. For the sample group, 1mL 2 mg/mL defatted Antarctic krill or AKP solution prepared with 20 mM phosphate buffer saline (PBS) (pH=7.0) was mixed with 20 μL BPB solution. While for the blank group, the equivalent buffer was used instead of the AKP solution. The mixture was centrifuged at 5000 rpm for 10 min, and the absorbance of collected supernatant was measured at 595 nm by a microplate reader (SpectraMax 190, Molecular Devices, San Jose, CA, USA). The surface hydrophobicity was obtained *via* the formula as follows (Eq. (3):

(3) $$\mathrm{Surfacehydrophobicity}\left(\mu gBPB/g\right)=20\times \left(A_{b}-A_{s}\right)/A_{b}$$

where *A_b_* and *A_s_* were the absorbances of blank and sample groups, respectively.

### 2.8 Molecular weight (MW) distribution assessment

As described by Zhang et al. [27] with some modifications, the MW distribution was assessed by an ultrafiltration-based method. The AKP powders were redissolved with deionized water and homogenized (T18 Basic, IKA Instrument and Equipment Co., Ltd., Guangzhou, China) at 10,000 rpm for 1 min. Then the solutions were isolated by sequential ultrafiltration and centrifugation using Amicon Ultra-15 centrifugal filters (Millipore, Bedford, MA, USA) with 10 kDa, 3 kDa, and 1 kDa MW cut-offs at 10,000 rpm for 30 min. All recovered fractions (<1 kDa, 1-3 kDa, 3-10 kDa, and >10 kDa) were freeze-dried and weighted. The MW distribution was calculated and expressed as the weight percentage of each isolated fraction.

### 2.9 Amino acid composition analysis

Following the procedures of Su et al. [3] and Fan et al. [5] with minor modifications, the amino acid composition was analyzed by a LA8080 automated amino acid analyzer (Hitachi Co., Ltd., Tokyo, Japan). The defatted Antarctic krill or AKP powders were transferred into headspace vials and hydrolyzed with 6 M HCl solution under a nitrogen atmosphere at 110 ^o^C for 22 h. Afterward, the hydrolysates were dried under a nitrogen stream, redissolved with deionized water, and filtered through 0.22 μm microfiltration membranes (BKMAM Biotechnology Co., Ltd., Changde, China) for the analysis. The amino acid composition was calculated and expressed as the molar percentage.

### 2.10 Scanning electron microscopy (SEM)

The micromorphology was observed by a TM3000 SEM (Hitachi Co., Ltd., Tokyo, Japan) as described by Zhang et al. [28] with some modifications. The defatted Antarctic krill or AKP powders were sprayed with gold through an ion-sputtering coating instrument prior to the observation. The SEM photographs were acquired at an accelerating voltage of 10 kV and a magnification of 400. Besides, the optical photographs were also obtained through a smartphone camera for the comparison of appearances.

### 2.11 Label-free peptidomic analysis based on HPLC-MS/MS

The label-free peptidomic analysis based on HPLC-MS/MS was performed following the procedures of Zhang et al. [29] with some modifications. The experiment was carried out using a times TOF pro high-resolution mass spectrometer (MS) system coupled with a Nano Elute high-performance liquid chromatograph (HPLC) (Bruker, Billerica, MA, USA). Briefly, the flavourzyme-hydrolyzed AKP (F-AKP, at 0 W) or high-intensity ultrasound-assisted flavourzyme-hydrolyzed AKP (HUF-AKP, at 400 W) sample was desalted through an Empore SPE C18 Cartridge (Sigma-Aldrich, St. Louis, MO, USA) and then concentrated by vacuum centrifugation (Concentrator Plus, Eppendorf AG Co., Ltd., Hamburg, Germany). Then the sample was loaded onto a C18 trap column (5 µm, 100 µm×2 cm, Thermo Fisher Scientific) in 0.1% formic acid/water solution, and eluted over a C18 reversed-phase analytic EASY column (1.9 µm, 75 µm×25 cm, Thermo Fisher Scientific) with a linear gradient of 0.1% formic acid/84% acetonitrile solution at a 300 nL/min flow rate. The MS/MS data were obtained in the parallel accumulation serial fragmentation mode using a time-of-flight analyzer at the positive ion mode, 1.5 kV ion source voltage, 0.6-1.6 Vs/cm^2^ ion mobility, 100-1700 m/z mass range, 10 MS/MS scans per one full MS scan, and 24 s dynamic exclusion time. The raw MS/MS data were further analyzed by the Peaks software for peptide identification.

The identified peptide sequences were searched against the UniProt Knowledgebase (https://www.uniprot.org/, accessed April 29, 2026) with unspecific enzymes, non-fixed modifications, and variable modifications of N-terminal acetylation and Met oxidation. Sequences with an abundance >10^5^ in MS/MS spectra were considered as enriched peptides and selected for the further analysis. Meanwhile, the Bitter Peptide Prediction (BPP) tool of Database of Food-derived Bioactive Peptides (DFBP) (http://www.cqudfbp.net/bitterPrediction/tools/DataInput.jsp, accessed July 3, 2026) was utilized to calculate the Q values of identified peptides, which represented their average hydrophobicities. Furthermore, following the methods of Ye et al. [30] and Mei et al. [31] with slight modifications, the machine learning-based Taste Peptides Decision Model (TPDM) of TastePeptides-Meta Database (https://tastepeptides-meta.com/TPDM, accessed July 10, 2026) was applied for the virtual screening of potential bitter and umami peptides. Specifically, peptides predicted as bitter with a non-bitter probability of <0.50 were considered bitter candidates, whereas those identified as umami with a probability of >0.95 and simultaneously non-bitter were regarded as umami candidates.

### 2.12 Statistical analysis

The taste sensory evaluation was performed with eight biological samples, whereas all the other experiments were carried out with three biological samples. For each biological sample, the technical analysis was conducted in triplicate. All Figures were drawn using the Origin V2021 software (Origin-Lab, Northampton, NC, USA) or Microsoft PowerPoint 2021 software. The analysis of variance (ANOVA) was conducted by the SAS V9.2 software (SAS Institute Inc., Carry, NC, USA). The principal component analysis (PCA), Pearson’s correlation analysis, and hierarchical cluster analysis (HCA) were performed with the plug-in applications in Origin V2021. The Duncan multiple range test was adopted to establish differences among groups, and the statistical significance was considered as *p*<0.05.

## 3 Results and Discussion

### 3.1 Effects of HUF on protein recovery and DH of AKPs

**Fig. 1:**
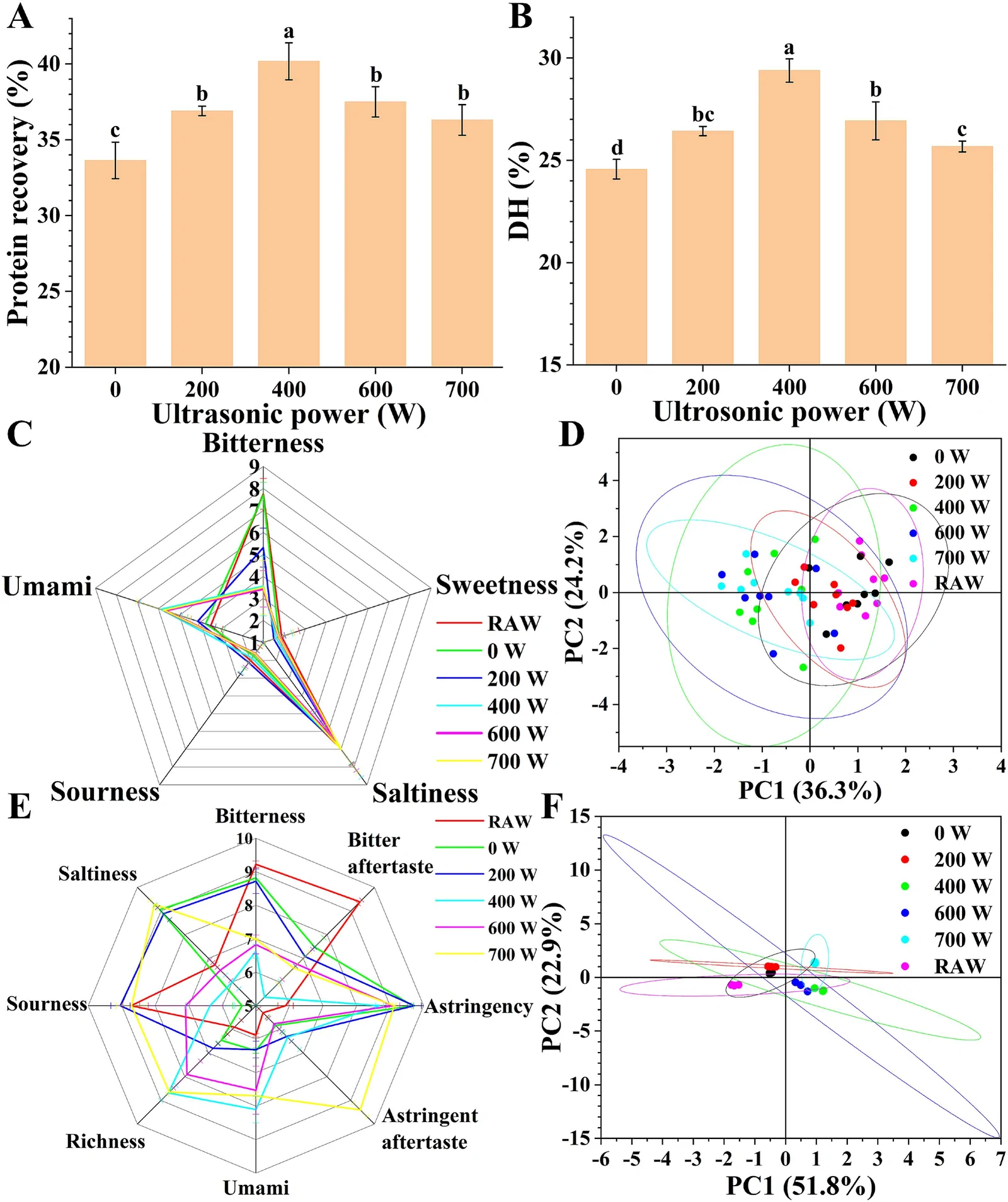
Protein recovery (A), DH (B), taste sensory quality (C-D), and E-tongue perception (E-F) of AKPs prepared through HUF with different ultrasonic powers. Different lowercases above the error bar in A-B indicated significant differences among various samples (*p* < 0.05). D and F show the PCA plots of sensory quality (D) and E-tongue perception (F), respectively. DH, degree of hydrolysis; RAW, defatted Antarctic krill; E-tongue, electronic tongue; PCA, principal component analysis.

**Fig. 2:**
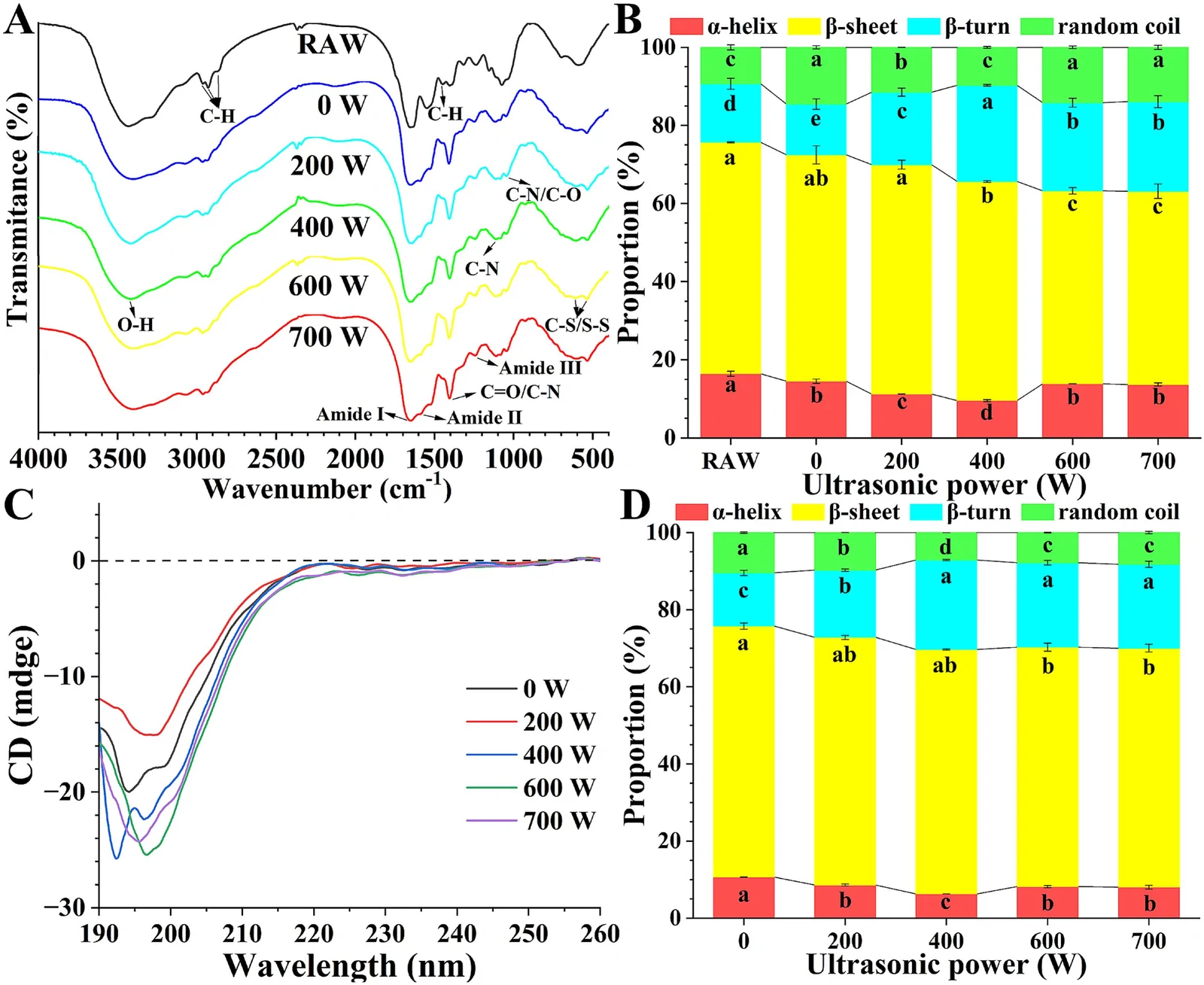
Secondary structure of AKPs prepared through HUF with different ultrasonic powers based on FT-IR and CD spectroscopy. A-B, FT-IR spectra (A) and calculated secondary structures (B), respectively; C-D, CD spectra (C) and calculated secondary structures (D), respectively. Different lowercases above the error bar in B and D indicated significant differences among various samples at the same secondary structure (*p* < 0.05). RAW, defatted Antarctic krill; FT-IR, Fourier transform-infrared; CD, circular dichroism.

**Fig. 3:**
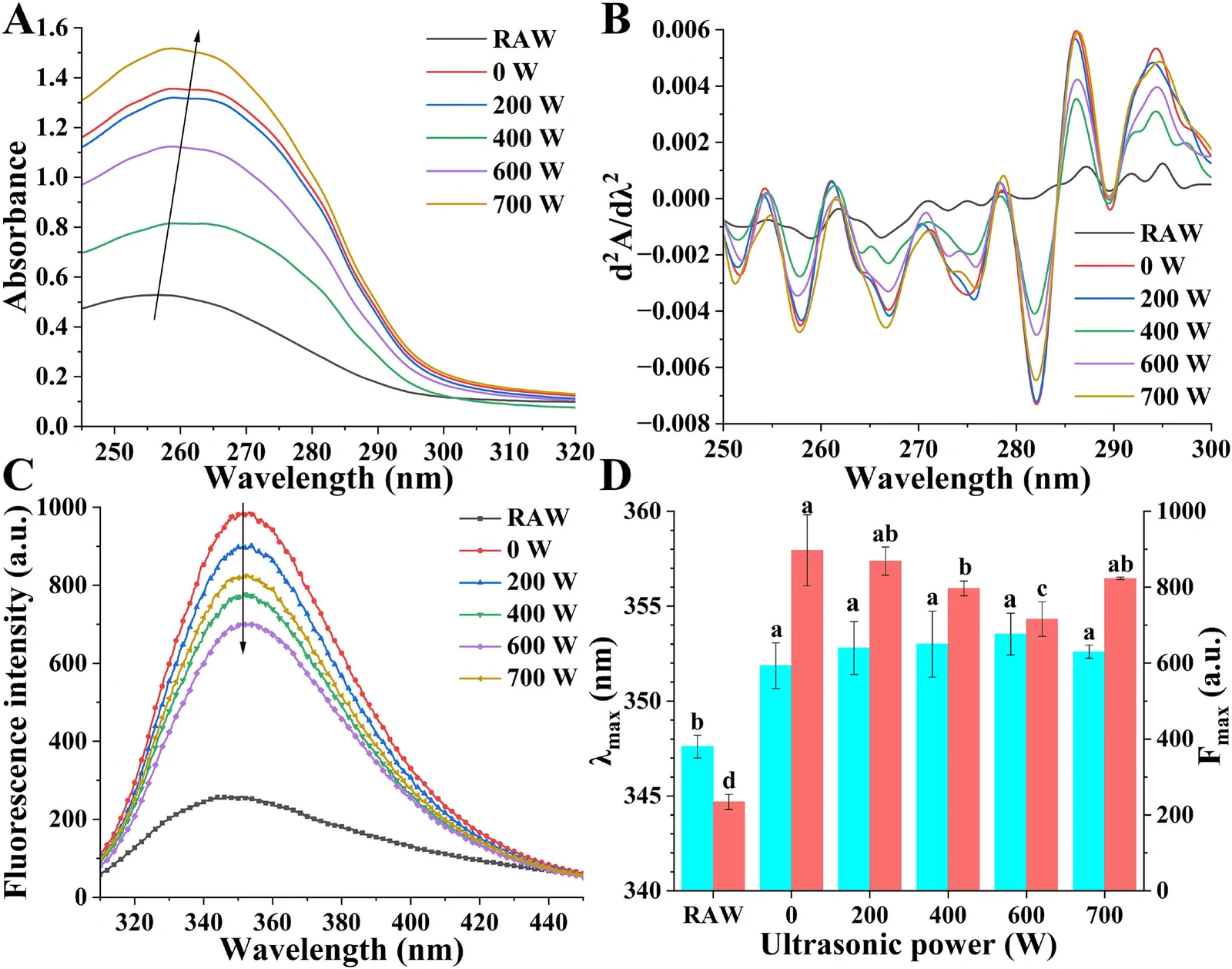
Tertiary structure of AKPs prepared through HUF with different ultrasonic powers based on UV–vis and intrinsic fluorescence spectroscopy. A-B, UV–vis spectra (A) and second derivative spectra (B), respectively; C-D, intrinsic fluorescence spectra (C) and maximum fluorescence intensities (*F_max_*) along with corresponding wavelengths (*λ_max_*) (D), respectively. Different lowercases above the error bar in D indicated significant differences among various samples at the same parameter (*p* < 0.05). RAW, defatted Antarctic krill; UV–vis, ultraviolet–visible.

The protein recovery and DH of AKPs prepared by HUF with different ultrasonic powers are illustrated in Fig. 1A and Fig. 1B, respectively. As the ultrasonic power of HUF rose from 0 to 400 W, the protein recovery and DH of AKP gradually increased by approximately 6.54% and 4.83%, respectively (*p*<0.05). Consistently, Huang et al. [19] found that the DH of sweet apricot kernel peptides prepared through HUF was notably improved by about 5% as the ultrasonic power increased from 0 to 325 W. Li et al. [4] reported that the ultrasound-assisted treatment at 350 W for 30 min significantly increased the yield of basic electrolyzed or deionized water-extracted Antarctic krill proteins by around 9%. This can be mainly ascribed to the cavitation effect of ultrasound, which could facilitate the protein unfolding and hydrophobic cleavage sites exposure for enzymolysis (Fig. 2, Fig. 3), thereby improving the hydrolytic activity of flavourzyme [1], [19]. However, when the ultrasonic power further rose to 700 W, both protein recovery and DH of AKP gradually decreased to the levels of the sample treated at 200 W (*p*<0.05). Accordingly, Huang et al. [19] observed the dramatic DH decrease of sweet apricot kernel peptides during HUF when the ultrasonic power further elevated from 325 W to 585 W. This is probably attributed to two following reasons: on one hand, the hydroxyl radicals generated through violent cavitation of high-power ultrasound would destroy the structure or attack the active center of flavourzyme, resulting in the activity reduction [19]; on the other hand, over-unfolded protein molecules due to overhigh-power ultrasound may lead to the hydrophobicity-driven reaggregation, disturbing the protein dissolution and subsequent flavourzyme hydrolysis [32]. Overall, these findings indicated that high-intensity ultrasound promoted the protein degradation and peptide release during the flavourzyme hydrolysis of Antarctic krill.

### 3.2 Effects of HUF on taste characteristics of AKPs based on sensory and E-tongue evaluations

Fig. 1C shows the taste sensory quality of AKPs prepared by HUF with different ultrasonic powers. All the taste sensory parameters of defatted Antarctic krill were not obviously different from those of F-AKP (at 0 W) (*p*>0.05), implying that only flavourzyme hydrolysis didn’t markedly change the overall tastes of AKP. Additionally, the sensory scores of saltiness, sourness, and sweetness for all AKPs were not obviously different (*p*>0.05), but 400-700 W HUF contributed to 1.8-4.3 lower bitterness and 1.6-2.1 higher umami sensory scores of AKPs than 0-200 W HUF (*p*<0.05). Similarly, Siewe et al. [33] reported that the ultrasound-assistance at 30-45% amplitudes resulted in a remarkable bitterness reduction and umami increase for the sensory quality of natural fish flavouring compared to samples without ultrasonic treatment. The PCA of taste sensory quality was further conducted with the result presented in Fig. 1D. The first axis (PC1) could explain 36.3% of the total variation, and the first two axes (PC1+PC2) were responsible for 60.5%, suggesting acceptable separation and certain variations among the overall sensory tastes of these samples.

Moreover, the E-tongue analysis was also performed with the result illustrated in Fig. 1E. The E-tongue sourness and astringency aftertaste values of all samples were below the tasteless point, and their saltness values showed no apparent differences among varied groups (*p*>0.05), which were partially accordant with their taste sensory results (low sourness and similar saltness scores) (Fig. 1C). While for the other E-tongue parameters, 400-700 W HUF allowed AKPs to exhibit significantly lower astringency, bitterness, and bitterness aftertaste values as well as higher umami and richness values than 0-200 W HUF (*p*>0.05), which were also corresponding with part of their sensory characteristics (bitterness decrease and umami increase by 400-700 W HUF) (Fig. 1C). Additionally, the PCA of E-tongue data was also carried out with the result displayed in Fig. 1F. PC1 could explain 51.8% of the total variation, and PC1+PC2 can explain 74.7%, which were both greatly higher than those for their taste sensory scores, implying significant separations and variations among global E-tongue perceptions of different groups. In summary, the taste characteristics based on both sensory and E-tongue evaluations verified the bitterness-reducing and umami-enhancing influences of ≥400 W ultrasound on AKPs during HUF.

### 3.3 Effects of HUF on secondary structure of AKPs based on FT-IR and CD spectra

The chemical structures of AKPs prepared through HUF with different ultrasonic powers were determined by FT-IR, and the results are shown in Fig. 2A. The broad band at 3200-3600 cm^-1^ primarily showed the O-H stretching vibration of hydroxy groups and hydrogen bonds, while the weak peaks at around 2960, 2930, and 2870 cm^-1^ were associated with the C-H stretching vibration of protein/peptide skeleton [28]. Particularly, the bands at 1600-1700, 1540-1600, and 1200-1280 cm^-1^ were the characteristic peaks of proteins/peptides and associated with their secondary structures, representing amine I, II, and III bands, respectively, which showed the coupled vibration of C=O stretching, C-N stretching, and N-H bending [24]. Additionally, the bands at around 1450, 1400, 1120, and 1040 cm^-1^ mainly belonged to the vibrations of C-H bending, carboxylic C=O stretching, skeleton C-N stretching, and C-O stretching, respectively, whereas the bands at about 600 and 540 cm^-1^ may be mainly related to the C-S stretching vibration [24], [28]. Generally, the wavenumbers and strengths of these characteristic FT-IR bands were basically corresponding with previous reports of Yao et al. [34] on ultrasound-assisted alkaline-extracted Antarctic krill proteins and Fan et al. [5] on AKPs prepared by composite enzymolysis.

**Fig. 4:**
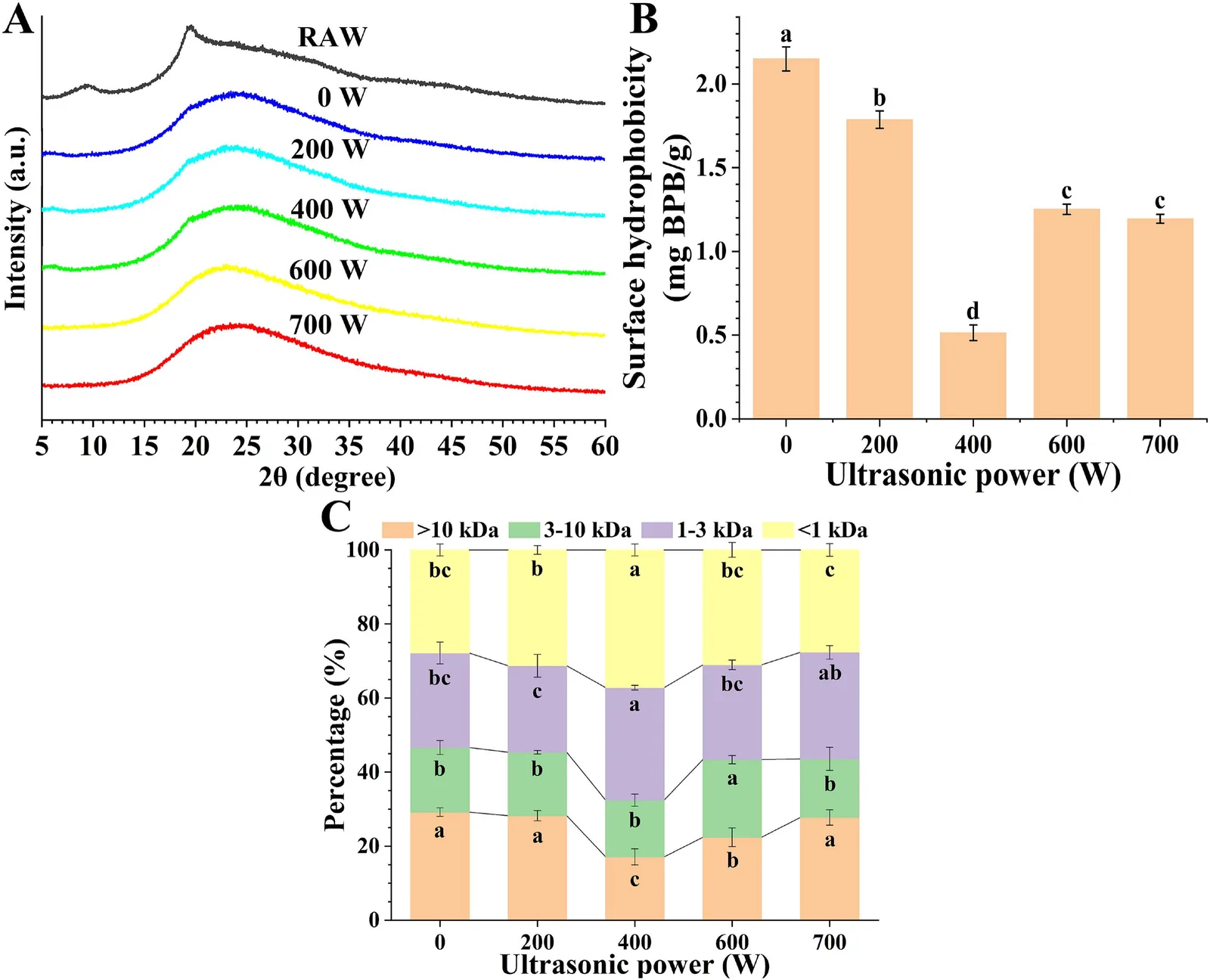
XRD spectra (A), surface hydrophobicity (B), and MW distribution (C) of AKPs prepared through HUF with different ultrasonic powers. Different lowercases below the error bar in B-C indicated significant differences among various samples at the same parameter (*p* < 0.05). RAW, defatted Antarctic krill; XRD, X-ray diffraction; MW, molecular weight.

The percentages of different secondary structures for defatted Antarctic krill and varied AKPs, including α-helix, β-sheet, β-turn, and random coil, were calculated based on the absorption value of amide I bands, and the results are shown in Fig. 2B. Overall, owing to the protein degradation under flavourzyme, all AKPs showed notably lower α-helix and higher β-turn contents than the defatted Antarctic krill (*p*<0.05). Meanwhile, during the power elevation of HUF from 0 to 700 W, the ratio of ordered secondary structures (α-helix + β-sheet) gradually declined from 72.48% to 63.26% (*p*<0.05), whereas the percentage of disordered secondary structures (β-turn + random coil) dramatically increased by 9.22% (*p*<0.05). The same change in secondary structures was reported by Vanga et al. [35] on pulsed ultrasound-treated almond milk proteins. The ultrasonic cavitation-induced strong shear forces and high pressure could break the hydrogen bond networks responsible for the extensibility maintenance of α-helix and β-sheet [16]. Meanwhile, the ultrasound-induced DH promotion (Fig. 1B) and resultant MW reduction (Fig. 4C) can be another important reason for the decreased contents of α-helix and β-sheet, since β-turn and random coil are more universal conformations distributed in smaller peptides [27]. Nevertheless, when the ultrasonic power increased from 400 W to 700 W, the β-turn content slightly reduced, whereas the α-helix content significantly increased (*p*<0.05). Correspondingly, Rafique et al. [36] found that 600 W ultrasound unexpectedly contributed to slightly higher α-helix but lower β-turn contents of oat protein isolates than 400 W ultrasound. Considering that >400 W HUF also led to the DH decline (Fig. 1B) and average MW increase (Fig. 4C) of AKPs, this can be associated with the overhigh-power ultrasound-induced hydrophobic reaggregation of proteins, which may disturb the α-helix degradation to β-turn by reducing the contact area between enzymes and proteins [32].

Furthermore, the CD spectra of AKPs prepared by HUF with different ultrasonic powers were detected with results illustrated in Fig. 2C. The fitting and calculation of CD spectra were then performed to further validate the conformational variations among different AKPs, and the results are presented in Fig. 2D. Generally, the same sample showed a slightly higher β-sheet percentage, less α-helix content, and lower random coil proportion than those calculated based on FT-IR (*p*<0.05). However, both of the secondary structure results derived from CD and FT-IR spectra demonstrated a similar tendency during the change of HUF power. In general, these results manifested that appropriate high-intensity ultrasound effectively promoted the AKP conformation conversion from extended and ordered to compact and disordered during flavourzyme hydrolysis.

### 3.4 Effects of HUF on tertiary structure of AKPs based on UV-vis and fluorescence spectra

The UV-vis and intrinsic fluorescence spectroscopy were performed to further analyze the effects of HUF powers on tertiary structures of AKPs. The UV-vis spectra of different AKP samples are illustrated in Fig. 3A. Generally, compared with the defatted Antarctic krill, all AKPs showed significantly higher absorbances within the UV range of 250-320 nm (*p*<0.05), and their maximum absorption wavelengths exhibited an obvious blue shift from 256 nm to 259 nm (*p*<0.05). Considering that phenyl ring groups of Phe, Trp, and Tyr were the main chromophores within this range [18], this result indicated that these residues buried in the highly hydrophobic core of Antarctic krill proteins were partially released to the AKP surface, which can be a vital bitterness source of AKPs since they are important bitter contributors [10]. Moreover, to further obtain the absorbance variations among different chromophores, the second derivative analysis was conducted on the UV-vis spectra with results presented in Fig. 3B. Among various maximum negative peaks of absorbances, Phe showed a characteristic peak at 257 nm and another peak at 268 nm, Tyr exhibited two related bands at 275 and 268 nm, while Trp had a major peak at 283 nm and a smaller one at 289 nm [37]. The second derivative absorbances of all these chromophores gradually reduced as the HUF power increased from 0 to 400 W (*p*<0.05), and then dramatically enhanced when the power was further augmented to 700 W (*p*<0.05).

**Fig. 5:**
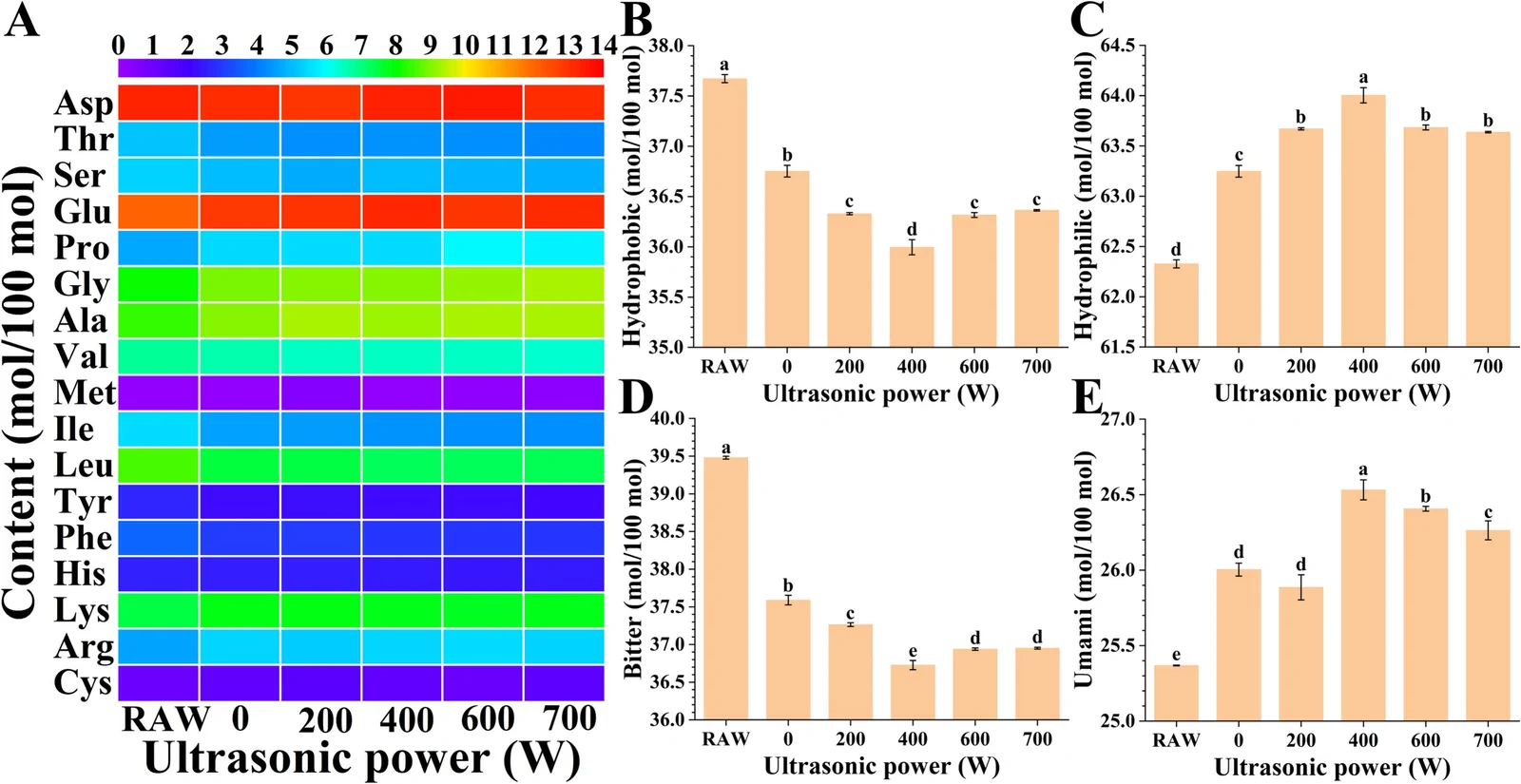
Amino acid composition of AKPs prepared through HUF with different ultrasonic powers. A, heatmap of content for each amino acid residue; B-C, total contents of hydrophobic (B) and hydrophilic (C) amino acid residues, respectively; D-E, total contents of bitter (D) and umami (E) amino acid residues, respectively. Different lowercases above the error bar in B-E indicated significant differences among various samples (*p* < 0.05). RAW, defatted Antarctic krill.

Besides, Fig. 3C shows the intrinsic fluorescence spectra of different AKP samples, and Fig. 3D presents their maximum fluorescence intensities along with corresponding wavelengths. The intrinsic fluorescence intensity of proteins/peptides is positively correlated with the Trp and Tyr residues exposed to water, thereby reflecting the alterations in tertiary structures [18]. Overall, the variation trend of all samples in intrinsic fluorescence spectra was similar to that in UV-vis spectra (Fig. 3A-B). All AKPs also displayed both higher intrinsic fluorescence intensities and blue-shifted maximum fluorescence wavelengths than the defatted Antarctic krill (*p*<0.05). More importantly, the intrinsic fluorescence intensity of AKPs also first declined and then rose during the power increase of HUF from 0 to 700 W (*p*<0.05), which was in line with their changes in DH (Fig. 1CB) and secondary structures (Fig. 2B & D). Consistently, Dong et al. [38] reported the reduction-then-increase changes of intrinsic fluorescence intensity for enzyme-extracted chicken bone proteins as the ultrasound-pretreated powder increased from 240 W to 400 W. Appropriate high-intensity ultrasound facilitated the conformational transition and protein unfolding (Fig. 2), promoting the enzymolytic efficiency (Fig. 1CB) and the exposure of buried hydrophobic residues, which allowed the flavourzyme to cleave more hydrophobic residues (Fig. 5A-B), probably contributing to the reduction of both UV-vis absorbances and intrinsic fluorescence intensities of HUF-AKPs (Fig. 3), and also becoming an important reason for the bitterness attenuation of these samples (Fig. 1C & E). Meanwhile, the absorbance or intensity increase induced by overhigh-power HUF might be relevant to the above-mentioned protein reaggregation and enzymolytic activity reduction [19], [32].

### 3.5 Effects of HUF on XRD spectra of AKPs

The crystal states of AKPs prepared by HUF with different ultrasonic powers were analyzed through XRD with the results shown in Fig. 4A. Briefly, the defatted Antarctic krill presented a main diffraction peak at *d*=4.54 Å (2*θ*=19.54^o^) and a weaker peak at *d*=9.24 Å (2*θ*=9.56^o^), which can be attributed to the interchain hydrogen bond distance and stacking distance between cross β-sheets, respectively [25]. Conversely, all AKPs exhibited a broad and diffuse peak at approximately *d*=3.54-3.86 Å (2*θ*=23.02-25.14^o^) without obvious differences among each other. Similar XRD spectra of collagen peptides from *Takifugu rubripes* bones were reported by Guo et al. [39], indicating an amorphous peptide structure with limited long-range molecular order, which may be mainly associated with β-turn and random coil. These results partially agreed with the difference between the defatted Antarctic krill and AKPs in secondary structures (Fig. 2B), and suggested that high-intensity ultrasound didn’t evidently change the crystalline structure of AKPs during HUF.

### 3.6 Effects of HUF on surface hydrophobicity of AKPs

Fig. 4B exhibits the surface hydrophobicity of AKPs prepared by HUF with different ultrasonic powers. Briefly, the surface hydrophobicity of AKPs gradually decreased by around 76.10% with the ultrasonic power increased from 0 to 400 W (*p*<0.05), and then markedly enhanced by approximately 1.33 times when the power further increased to 700 W (*p*<0.05). Consistently, Rafique et al. [36] found that the surface hydrophobicity of oat protein isolates was significantly reduced by 200-400 W ultrasound but could not be changed by 600 W ultrasound. Besides, this trend was obviously opposite to their DH and protein recovery changes (Fig. 1A-B) but in line with their variation in tertiary structure (Fig. 3). These findings indicated that appropriate high-intensity ultrasound could promote the exposure and cleavage of hydrophobic residues during flavourzyme hydrolysis to reduce bitterness and astringency of AKPs (Fig. 1C & E). In contrast, excessive ultrasonic power (>400 W) may disturb this progress, probably owing to Antarctic krill protein reaggregation and flavourzyme-hydrolytic activity reduction.

### 3.7 Effects of HUF on MW distribution of AKPs

The MW distributions of AKPs as affected by different ultrasonic powers of HUF were evaluated through ultrafiltration, and the results are illustrated in Fig. 4C. The ratio of large-MW fraction (>10 kDa) notably reduced from 29.20% to 17.12% (*p*<0.05) during the ultrasonic power increase from 0 to 400 W, whereas the content of small-MW fraction (<1 kDa) dramatically increased from 27.82% to 37.19% (*p*<0.05), suggesting an obvious average MW decline of AKPs due to the ultrasonic cavitation-induced DH promotion (Fig. 1B) [4], [19]. Accordantly, Quan et al. [17] investigated the effects of ultrasound-assisted enzymolysis with pepsin and trypsin on the MW distribution of oyster peptides, and found that 50-200 W ultrasound contributed to the significant increase of <3500 Da fraction and reduction of >8000 Da fraction. Conversely, during the further power increase to 700 W, the mass of <1 kDa in AKPs remarkably decreased to 27.65% (*p*<0.05), while the percentage of >10 kDa distinctly rose to 27.75% (*p*<0.05). This finding implied an unexpected average MW decline owing to DH reduction (Fig. 1B), which may be ascribed to protein reaggregation and enzymolytic activity reduction induced by the over-treatment of ultrasound [19], [32].

### 3.8 Effects of HUF on amino acid composition of AKPs

Fig. 5A illustrates the amino acid composition of AKPs prepared by HUF with different ultrasonic powers. In general, Asp and Glu were the most abundant amino acid residues of both defatted Antarctic krill and various AKPs (*p*<0.05), accounting for 12.93-13.56 mol% and 12.09-13.22 mol%, respectively, followed by Gly (8.06-9.30 mol%), Ala (8.37-9.34 mol%), Leu (7.32-8.55 mol%), and Lys (7.49-7.90 mol%) (*p*<0.05). Meanwhile, Met and Cys were two mostly deficient residues (*p*<0.05), which accounted for only 0.15-0.50 mol% and 1.09-1.48 mol%, respectively, followed by His (2.38-2.54 mol%) and Tyr (2.15-2.69 mol%) (*p*<0.05). Correspondingly, Fan et al. [5] reported the amino acid composition of AKPs hydrolyzed by papain and neutrase, and also found that Glu and Asp had the highest abundance, while Cys, His, Met, and Tyr showed the lowest molar percentage. Moreover, the effects of HUF powers on the total contents of hydrophobic (Ala, Val, Met, Ile, Leu, Pro, and Phe) and hydrophilic (Asp, Thr, Ser, Glu, Gly, Tyr, His, Lys, Arg, and Cys) residues in AKPs are revealed in Fig. 5B-C, respectively. Meanwhile, the changes in bitter (Phe, Gly, Ile, Leu, Met, Pro, Val, and Tyr) and umami (Asp and Glu) residue amounts in AKPs as affected by HUF powers are further illustrated in Fig. 5D-E, respectively.

The molar concentration of hydrophobic residues in varied samples followed an obvious order of defatted Antarctic krill > F-AKP (at 0 W) > HUF-AKPs (at 200-700 W) (*p*<0.05), whereas their hydrophilic residues content followed a completely reverse order (*p*<0.05). Besides, the variation in bitter residues content among different samples showed a trend almost the same as that in hydrophobic residues, since most bitter amino acids have a hydrophobic side chain [16]. The difference in umami residues amount among various groups was similar to that in hydrophilic residues, because two umami amino acids are both hydrophilic and accounted for the highest richness among all residues in defatted Antarctic krill or AKPs (Fig. 5A). These results were consistent with the sensory scores (Fig. 1C) and E-tongue perceptions (Fig. 1E) of bitterness and umami for these samples, implying that the alteration of amino acid composition can be an important mechanism of high-intensity ultrasound-induced debittering and umami-enhancing of AKPs during HUF. Specifically, ultrasonic cavitation promoted the exposure and subsequent enzymatic cleavage of buried hydrophobic/bitter residues (Figs. 3 & 4B), which simultaneously allowed AKPs to possess more hydrophilic/umami residues (Fig. 5A, C, & E), thereby contributing to the weakened bitterness and enhanced umami (Fig. 1C & E). Furthermore, HUF-AKP prepared at 400 W has the maximum hydrophilic/umami residues amount and minimum hydrophobic/bitter residues amount among all groups (*p*<0.05), which were in line with the DH (Fig. 1B), E-tongue perception (astringency, bitterness aftertaste, umami, and richness) (Fig. 1E), conformation (α-helix and β-turn contents) (Fig. 2B), tertiary structure (Fig. 3A-B), surface hydrophobicity (Fig. 4B), and MW distribution (Fig. 4C) results.

### 3.9 Effects of HUF on micromorphology and appearance of AKPs

**Fig. 6:**
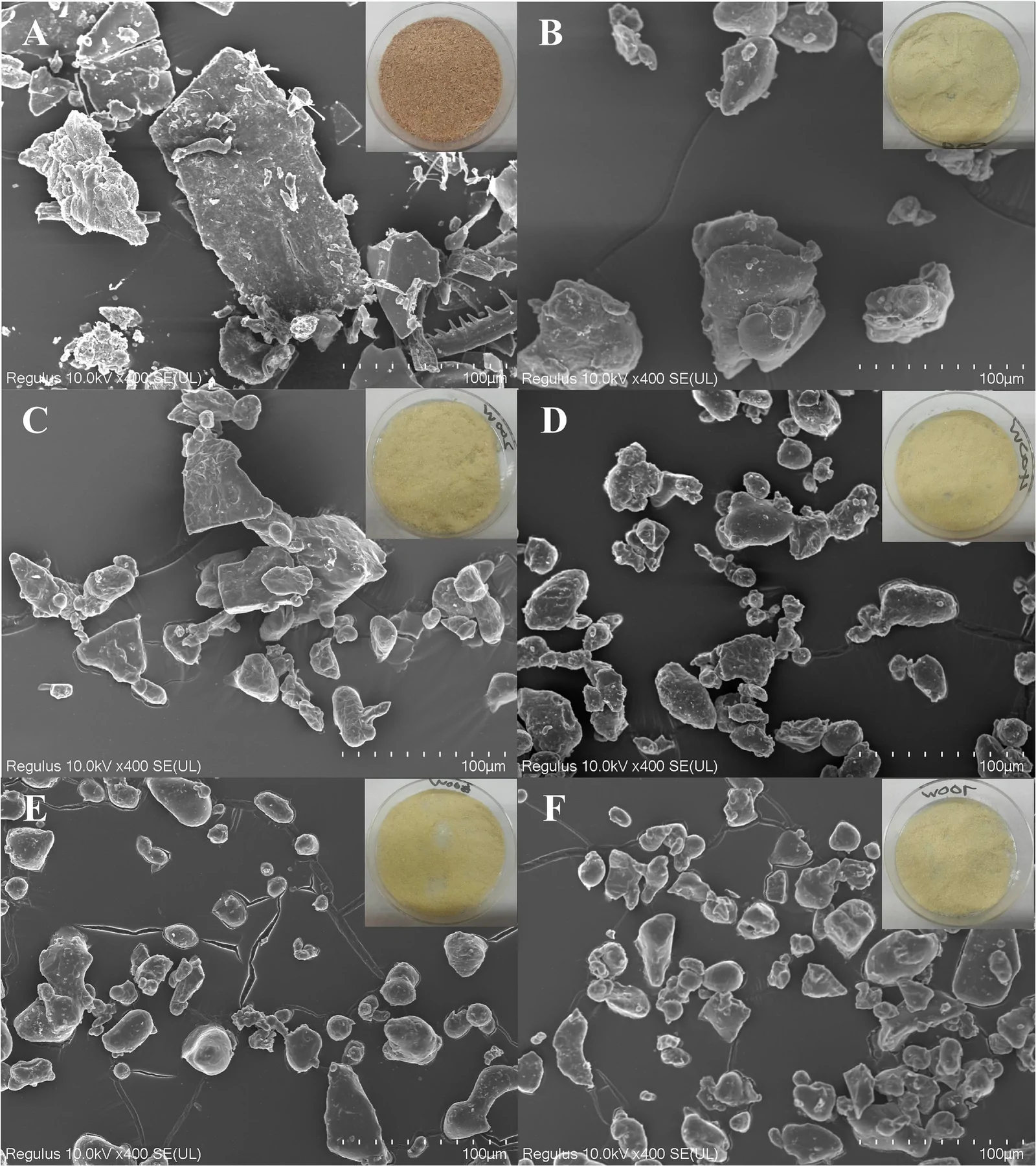
Micromorphologies (A1-F1) and appearances (A2-F2) of AKPs prepared through HUF with different ultrasonic powers. A, defatted Antarctic krill; B-F, AKP prepared through HUF. The ultrasonic powers of HUF: B, 0 W; C, 200 W; D, 400 W; E, 600 W; F, 700 W.

The microstructure and appearance of AKPs prepared by HUF with different ultrasonic powers are shown in Fig. 6A1-F1 and Fig. 6A2-F2, respectively. Generally, the defatted Antarctic krill was observed as large, ununiform, and irregular blocks with a brown color (Fig. 6A1 & A2). In contrast, all AKPs exhibited smaller, more even, and smoother aggregated particles with a similar light-yellow color (Fig. 6B1-F1 & B2-F2), owing to the degradation of proteins (Fig. 1B) and melanoidin-like chromophores [3]. More importantly, as the HUF power increased from 0 to 400 W, the particle morphology of AKP aggregates revealed an obvious trend of size reduction, outline smoothing, and shape regularization (Fig. 6B1-D1). Then the micromorphology and appearance of AKP aggregates no longer visibly changed after the HUF power exceeded 400 W during this process (Fig. 6D1-F1 & D2-F2). Consistently, Huo et al. [18] observed that 180 W ultrasound-assistance allowed the particles of walnut peptide aggregates through neutrase hydrolysis to be smaller, looser, and more uniform. The probable explanation can be associated with the ultrasound-induced protein structural unfolding and hydrophobic residues exposure through ordered conformations destruction (Fig. 2), which augmented the contact area between proteins and enzymes, thereby improving the enzymolysis (Fig. 1B) to produce greater numbers of small-MW peptides (Fig. 4C) [16], [18].

### 3.10 Multivariate statistical analyses based on PCA, Pearson’s correlation, and HCA

**Fig. 7:**
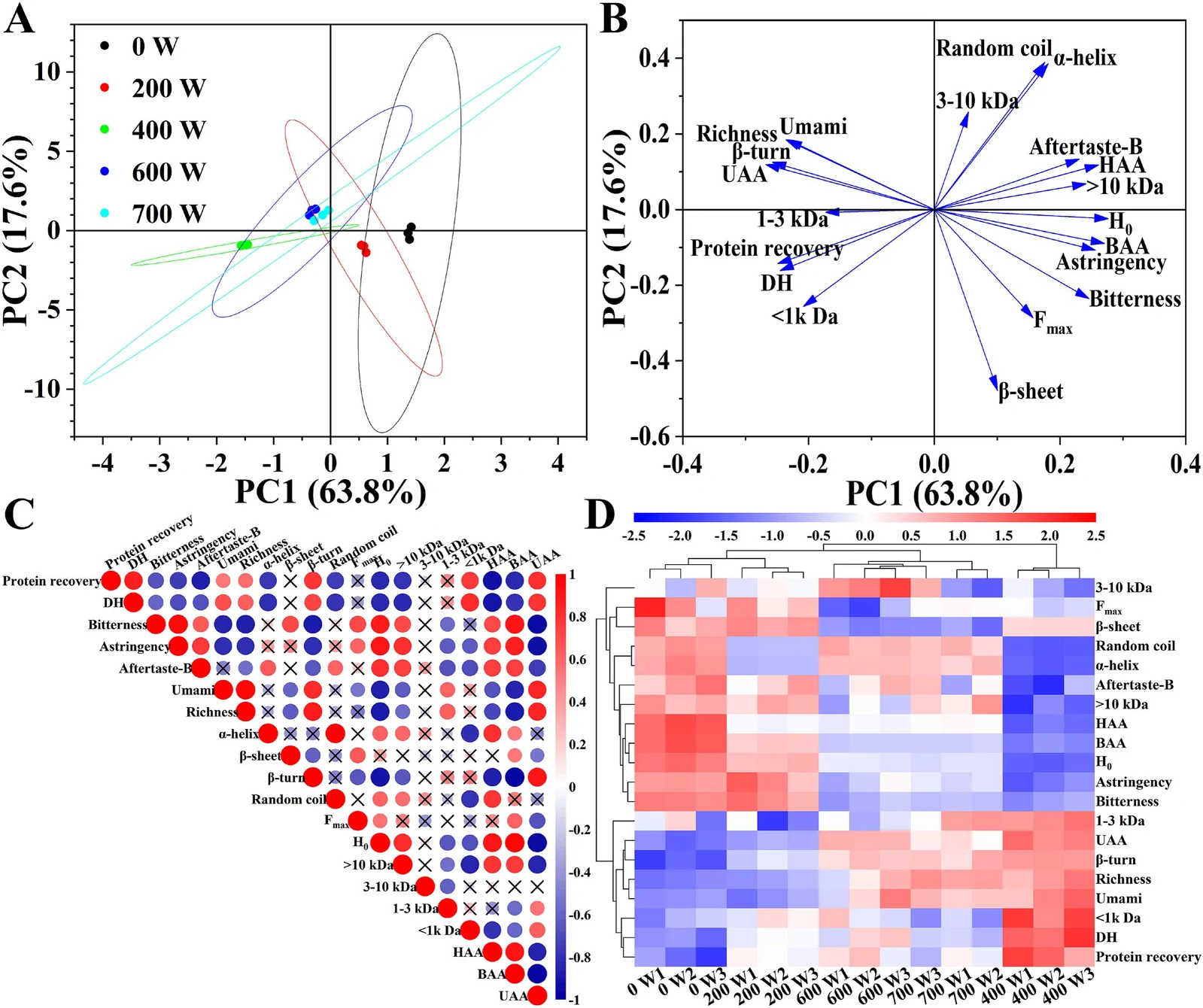
PCA (A-B), Pearson’s correlation analysis (C), and HCA (D) of significantly differential parameters for AKPs prepared through HUF at different ultrasonic powers. A and B show the score and loading plot for PCA, respectively. The color gradation and circle size in C correspond to the correlation coefficient (R value), while the pattern without an “×” mark represents a significant correlation (*p* < 0.05). The color gradation in D represents the Z-scores of corresponding parameters. The data of secondary structure and taste perception were from FT-IR spectra and E-tongue analyses, respectively. Aftertaste-B, bitter aftertaste; *F_max_*, maximum fluorescence intensity; *H_0_*, surface hydrophobicity; HAA, BAA, and UAA, contents of hydrophobic, bitter, and umami amino acid residues, respectively; PCA, principal component analysis; HCA, hierarchical cluster analysis.

To elucidate the underlying mechanism of high-intensity ultrasound-induced debittering and umami-enhancing of AKPs during HUF, the multivariate statistical analyses on significantly differential parameters were further performed with results displayed in Fig. 7. The PCA result exhibited that 63.8% of the total variation was explained by PC1 and 81.4% by PC1+PC2 (Fig. 7A-B), indicating notable variations among different AKPs and strong interrelations among various differential parameters. Meanwhile, agreeing with the PCA result (Fig. 7B), the Pearson’s correlation matrix (Fig. 7C) presented significant interrelations between each other of protein recovery, DH, bitter/umami perceptions, secondary/tertiary structures, MW distributions, and bitter/umami (hydrophobic/hydrophilic) residue compositions (excluding >10 kDa proportion), which mostly exhibited good significances (*p*<0.05) and strong correlations (|r|>0.80). Besides, all differential parameters in the HCA heat map (Fig. 7D) can be dispersed into two clusters (Euclidean distance ≤5), consistent with the loading plot of PCA (Fig. 7B). Specifically, Cluster 1 was composed of large-MW proportion (3-10 kDa and >10 kDa), tertiary structural parameters (*F_max_* and surface hydrophobicity), contents of most conformations (β-sheet, random coil, and α-helix), bitterness-associated tastes (bitterness, bitter aftertaste, and astringency), and bitter/hydrophobic residues percentage, which were mainly reduced or weakened by high-intensity ultrasound. In contrast, Cluster 2 consisted of small-MW proportion (<1 kDa and 1-3 kDa), β-turn proportion, umami-associated perceptions (umami and richness), umami residues percentage, and enzymolytic efficiency indexes (DH and protein recovery), which were primarily increased or strengthened through high-intensity ultrasound. Moreover, AKPs were classified into four groups (Euclidean distance <4) with general structural and taste properties following the order of Group (1) (F-AKP at 0 W) < Group (2) (HUF-AKP at 200 W) < Group (3) (HUF-AKPs at 600 W and 700 W) < Group 4 (HUF-AKP at 400 W), which corresponded with the score plot of PCA (Fig. 7A).

Moreover, based on these multivariate statistical findings (Fig. 7) and associated previous reports, the underlying mechanism can be preliminarily elucidated as follows. Primarily, the strong shear force and high pressure generated by appropriate high-intensity ultrasound promoted the conformational transition from ordered (α-helix and β-sheet) to disordered (mainly β-turn) (Fig. 2) through breaking H-bond networks, facilitating the structural unfolding of Antarctic krill proteins and the exposure of hydrophobic residues buried in the core, which improved the DH (Fig. 1B) and protein recovery (Fig. 1A) by augmenting the protein-enzyme contact area [4], [16], [18]. More importantly, this provided more hydrophobic cleavage sites for flavourzyme hydrolysis (Figs. 3 & 4B), resulting in the decline of average MW (>10 kDa proportion reduction and <3 kDa proportion increase) (Fig. 4C), the decrease of hydrophobic/bitter residues (especially Phe, Ile, Leu, Met, Pro, and Val) content (Fig. 5A, B, & D), and the relatively increase of hydrophilic/umami residues (mainly Asp and Glu) concentration (Fig. 5A, C, & E), which could directly contribute to the bitterness-associated perceptions weakening and umami-related tastes enhancement (Fig. 1C & E) [10], [19], [30]. Conversely, the overhigh-power ultrasound may induce the hydrophobicity-driven protein reaggregation, which disturbed α-helices degradation to β-turns (Fig. 2) and was not conducive to hydrophobic residues exposure for flavourzyme hydrolysis (Figs. 3 & 4B), thus decreasing the enzymolytic efficiency (Fig. 1A-B) due to protein-enzyme contact areas reduction. This further led to the average MW enlargement (Fig. 4C), hydrophobic/bitter residues amount raising (Fig. 5B & D), and hydrophilic/umami residues number diminishing (Fig. 5C & E), thereby resulting in the bitter enhancement and umami decline of AKPs (Fig. 1C & E) [32], [38], [40]. Meanwhile, 400 W was validated as the optimum HUF power to prepare bitterness-reduced and umami-enhanced AKPs based on the PCA and HCA results (Fig. 7A & D). Therefore, the HUF-AKP prepared at 400 W was selected for further investigation.

### 3.11 Peptide profiling by HPLC-MS/MS-based label-free peptidomics

**Fig. 8:**
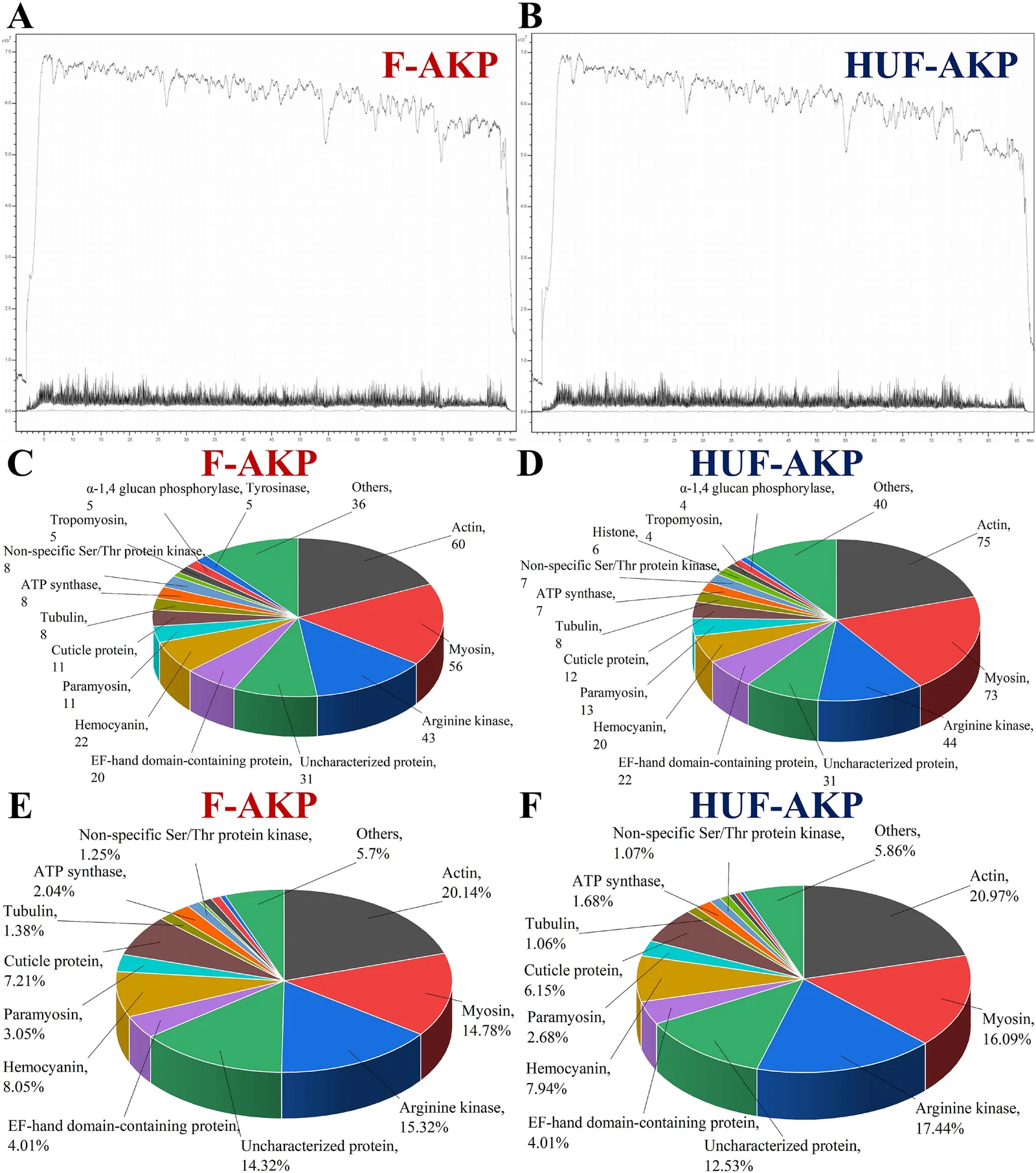
Total ion chromatograms of peptidomic analysis (A-B) and precursor protein distributions of enriched peptides (C-F) identified from F-AKP and HUF-AKP. C-D show the number distribution of identified peptides, while E-F display the relative abundances of identified peptides. Samples and ultrasonic powers: A, C, and E, F-AKP without ultrasound-assistance; B, D, and F, HUF-AKP prepared at 400 W.

The peptide profiles of F-AKP (at 0 W) and HUF-AKP (at 400 W) were obtained through *de novo* sequencing by HPLC-MS/MS-based label-free peptidomics for a comparative study. The total ion chromatograms of F-AKP and HUF-AKP are presented in Fig. 8A and Fig. 8B, respectively. 332 and 369 enriched peptides with the abundance in MS/MS spectra >10^5^ were acquired from F-AKP and HUF-AKP, respectively, among which 272 peptides were found in both samples (Fig. 11A). Their number and abundance distributions of protein source are illustrated in Fig. 8C-D and Fig. 8E-F, respectively. Briefly, myofibrillar protein components (mainly myosin, actin, paramyosin, and tropomyosin) were the most abundant protein source of these peptides and accounted for around 40% of total peptide number/abundance in both two samples, followed by various enzymes (mainly arginine kinase, ATP synthase, non-specific serine/threonine protein kinase, α-1,4 glucan phosphorylase, and tyrosinase), which accounted for about 20% of total number/abundance. Moreover, with the application of 400 W high-intensity ultrasound, the number percentage of myofibrillar protein-derived peptides expanded from 39.76% to 44.72%, and their relative abundance increased from 38.92% to 40.37%. This finding suggested that high-intensity ultrasound primarily facilitated the degradation of structural proteins rather than functional proteins in Antarctic krill.

**Fig. 9:**
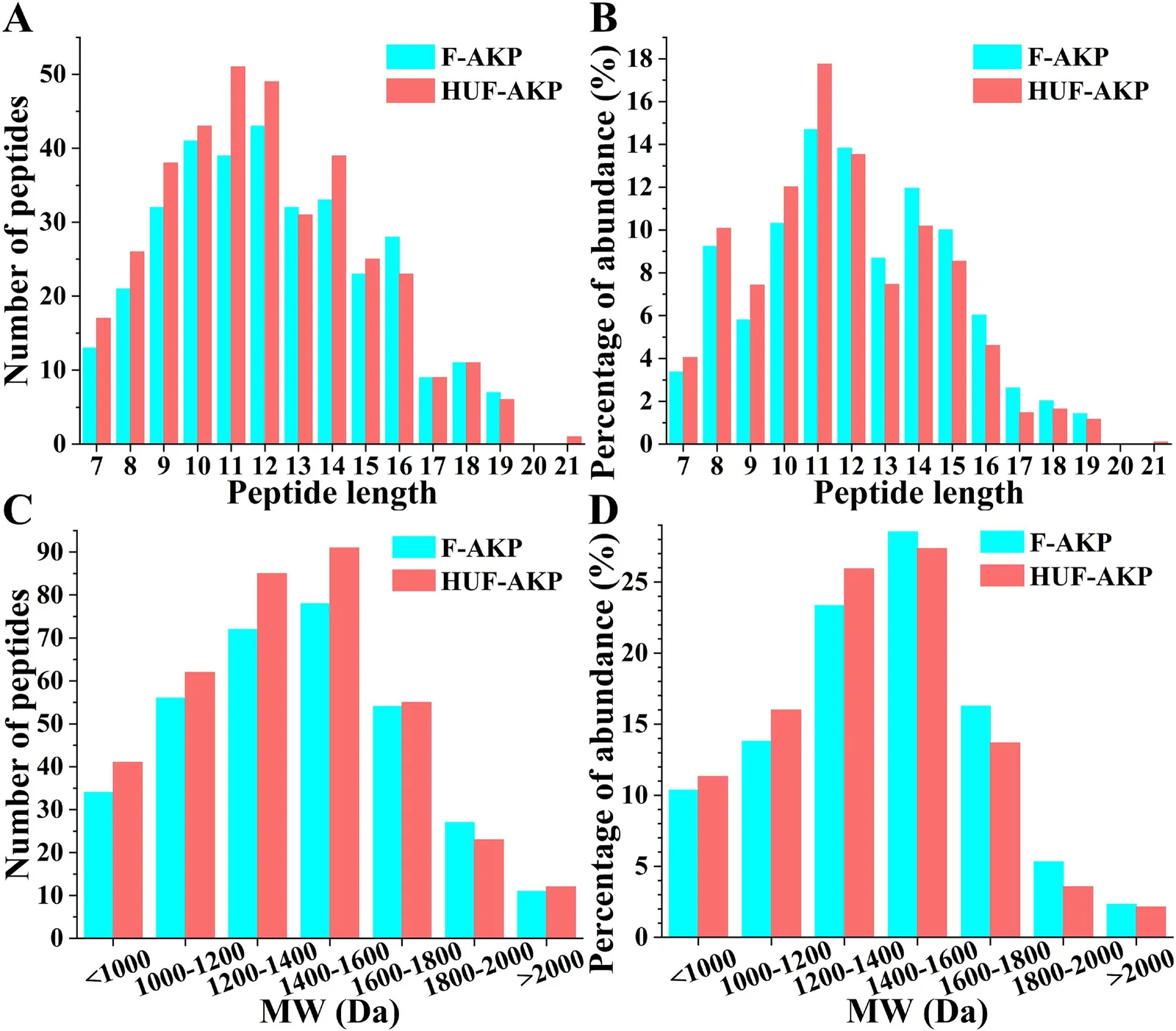
Length (A-B) and MW (C-D) distributions of enriched peptides identified from F-AKP and HUF-AKP by peptidomics. A and C show the number distribution of identified peptides, while B and D display the relative abundances of identified peptides. MW, molecular weight.

Moreover, their number/abundance distributions of peptide length and MW are displayed in Fig. 9A-B and Fig. 9C-D, respectively, which roughly demonstrate an overall Gaussian distribution. All peptides in both samples contained 7-21 residues with a MW of around 780-2300 Da, among which undecapeptides or dodecapeptides were the most abundant, constituting 12.95-13.82% of the total number with a relative abundance of 14.70-17.75%. Besides, for the F-AKP, only 56.93% peptides had ≤12 residues, which showed a relative abundance of 57.26%, and only 48.80% peptides possessed a MW of ≤1400 Da, possessing a relative abundance of 47.53%. Whereas for the HUF-AKP, over 60% peptides contained ≤12 residues, accounting for 64.84% of the total abundance, and the MW of over half of these peptides was ≤1400 Da, which comprised 53.24% of the total abundance. Similarly, Xiang et al. [12] reported that the MW of 0.5-3 kDa and length of 5-23 residues were the most important properties of enzymatically debittered cheese flavoring peptides, and the MW/length-reduction within this range usually leads to the bitterness attenuation. These findings implied that high-intensity ultrasound promoted Antarctic krill proteins to be degraded into smaller peptides during HUF, which can be a great contributor to the relatively lower bitterness and higher umami of HUF-AKP (Fig. 1C & E).

**Fig. 10:**
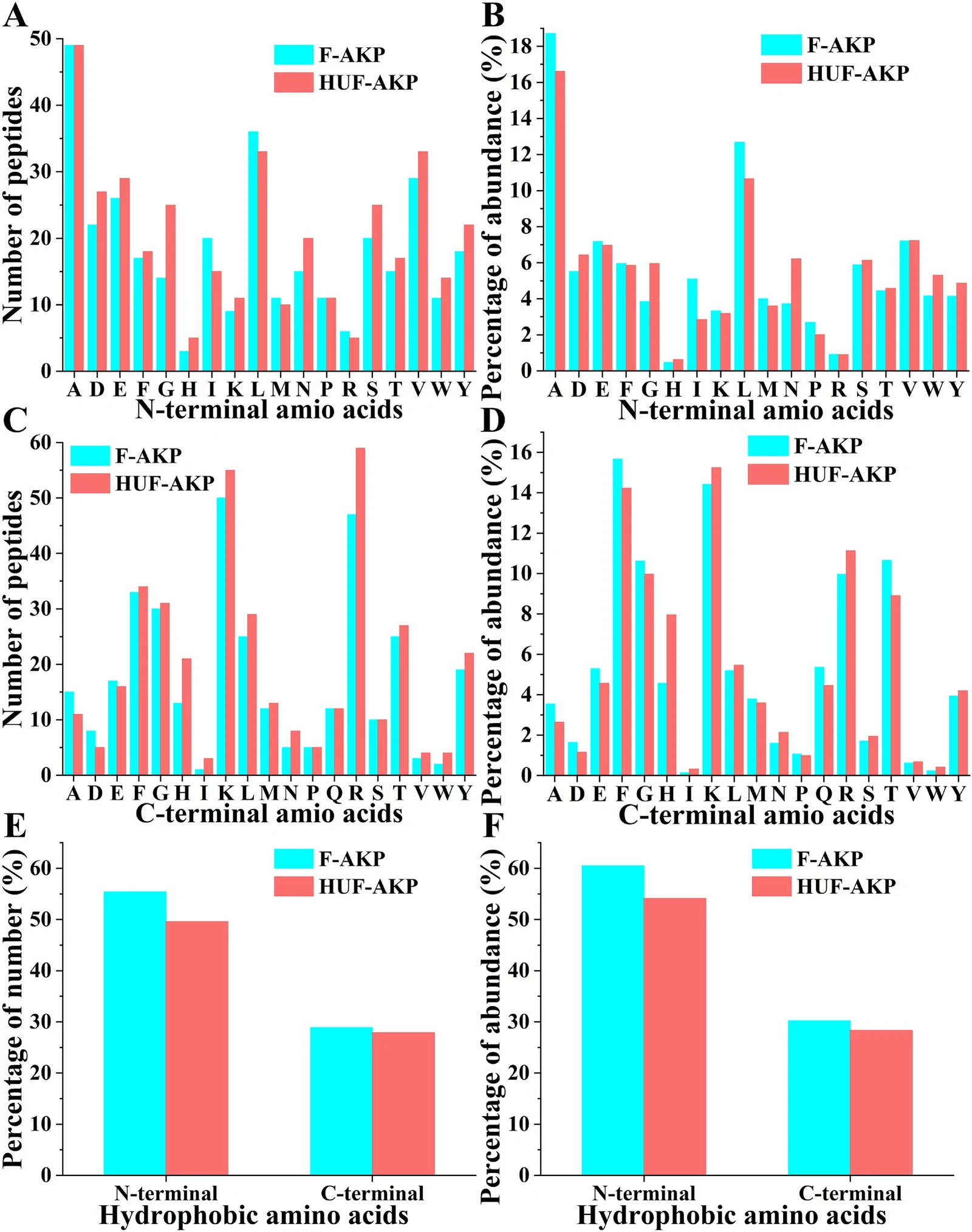
Terminal amino acid distributions of enriched peptides identified from F-AKP and HUF-AKP by peptidomics. A-B, N-terminal amino acid distribution of identified peptides; C-D, C-terminal amino acid distribution of identified peptides; E-F, hydrophobic amino acid content of N-terminus and C-terminus for identified peptides. A, C, and E show the number distribution of identified peptides, while B, D, and F display the relative abundances of identified peptides.

Additionally, the characteristic of terminal residues is commonly another important factor that affects the overall bitterness of peptides [11], [30], [41]. Fig. 10 shows the profile of N-terminal and C-terminal residue distributions for F-AKP and HUF-AKP. As presented in Fig. 10A-B, their most common residue at N-terminus was Ala (13.28-14.76% of the total number with a relative abundance of 16.62-18.72%), followed by Leu (8.94-10.84% of the total number with a relative abundance of 10.65-12.68%) and Val (8.73-8.94% of the total number with a relative abundance of 7.21-7.73%). While for the C-terminal residues, the most numerous ones were Lys (14.91-15.06%) and Arg (14.16-15.99%), but the most abundant ones were Phe (14.23-15.68%) and Lys (14.42-15.25%), followed by Gly (9.97-10.62%), Arg (9.97-11.12%), and Thr (8.90-10.65%) (Fig. 10C-D). More importantly, Yu et al. [11] found that N-terminal and C-terminal hydrophobic residues were two key attributes of milk-derived bitter peptides through peptidomics and machine learning, and Xiang et al. [12] also found that they were significant contributors to the bitterness of cheese flavoring peptides. As illustrated in Fig. 10E-F, HUF-AKP showed a N-terminal hydrophobic residue proportion of 5.83% lower in peptide number and 6.41% lower in relative abundance, respectively, than those of F-AKP. Meanwhile, compared with F-AKP, HUF-AKP allowed the hydrophobic residue number at the C-terminus to decline by 1.00% and its relative abundance to reduce by 1.91%. These results demonstrated that high-intensity ultrasound could promote the enzymatic cleavage at N-terminal and C-terminal hydrophobic residues *via* flavourzyme to reduce AKPs’ bitterness.

**Fig. 11:**
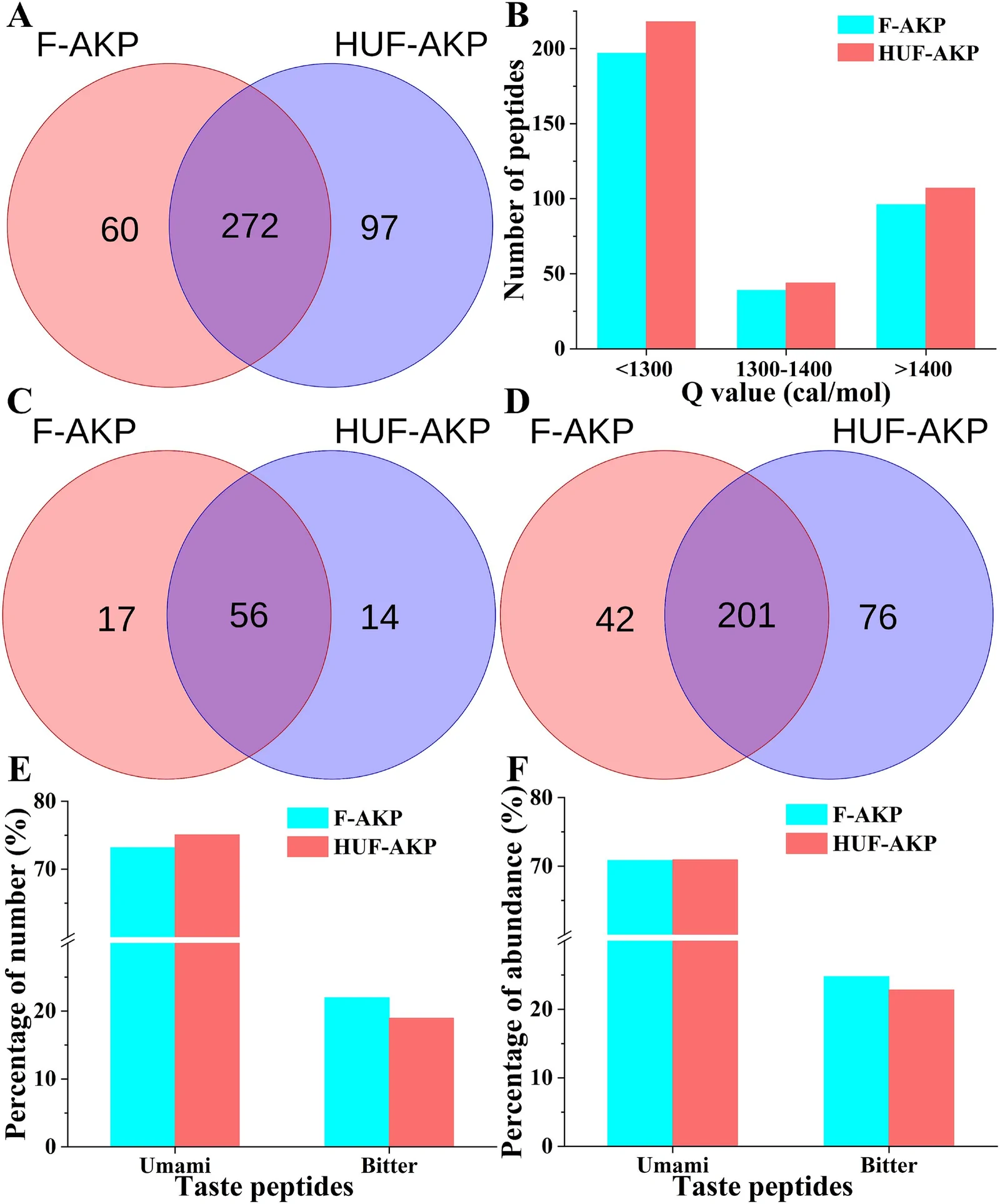
Distributions of potential bitter and umami peptides among enriched peptides identified from F-AKP and HUF-AKP by peptidomics. A, Venn diagram for identified peptides; B, Q value distribution of identified peptides; C, Venn diagram for predicted bitter peptides; D, Venn diagram for predicted umami peptides; E, percentage of numbers for predicted bitter and umami peptides; F, relative abundances for predicted bitter and umami peptides.

Besides, the average hydrophobicity is also considered with a high contribution degree to the overall taste of peptides by previous reports [10], [16], [42]. The peptide number distribution of Q values for both samples is presented in Fig. 11B. However, it did not show an obvious difference between F-AKP and HUF-AKP, implying that the average hydrophobicity of peptides wasn’t associated with the ultrasound-induced bitterness and umami alterations of AKPs during HUF. Furthermore, potential bitter and umami peptide constituents in two AKPs were predicted based on machine learning, and the results are illustrated in Fig. 11C-F. Briefly, HUF-AKP presented 3.02% lower number proportion of underlying bitter peptides and 1.87% higher number percentage of potential umami peptides than F-AKP (Fig. 11C-E). Meanwhile, although the potential umami peptides constituted a similar relative abundance in both samples, HUF-AKP still showed a 1.98% lower relative abundance of underlying bitter peptides than F-AKP (Fig. 11F). Correspondingly, Ye et al. [30] reported that 500 W high-intensity ultrasound allowed the high-pressure-treated bovine bone to release more umami peptides. These results suggested that high-intensity ultrasound could probably inhibit the generation of bitter peptides and expedite the release of umami peptides from Antarctic krill proteins during HUF.

## 4 Conclusions

This work unlocked the effects and underlying mechanism of high-intensity ultrasound-induced taste improvement of AKPs during HUF by integrating E-tongue, multi-spectroscopy, and label-free peptidomics. Results showed that ≤400 W high-intensity ultrasound promoted ordered conformations (α-helices and β-sheets) to convert into disordered conformations (mainly β-turns), thus facilitating the unfolding of structural proteins (mainly myofibrillar proteins) and cleavage of exposed hydrophobic residues (reduction of UV-vis absorbance, intrinsic fluorescence intensity, and surface hydrophobicity) during enzymolysis, which improved the DH and protein recovery. These changes contributed to the decline of average MW, peptide length, hydrophobic/bitter residues content, and N-terminal/C-terminal hydrophobic residues proportion of AKPs, which resulted in the generation of fewer underlying bitter peptides, thereby weakening bitterness-associated sensory or E-tongue perceptions (bitterness, bitter aftertaste, and astringency). Meanwhile, the hydrophilic/umami residues amount in AKPs was relatively increased, resulting in the release of more potential umami peptides, contributing to the umami-related taste (umami and richness) enhancement. Nevertheless, >400 W high-intensity ultrasound disturbed α-helices degradation along with subsequent exposure and cleavage of hydrophobic residues (increased UV-vis absorbance, intrinsic fluorescence intensity, and surface hydrophobicity during enzymolysis, which decreased the DH and protein recovery, further leading to the average MW enlargement, hydrophobic/bitter residues amount increase, and hydrophilic/umami residues number decrease, thus resulting in bitterness enhancement and umami decline of AKPs. This study could help understand the taste-improving mechanism of high-intensity ultrasound on food-derived peptides and provide practical support to the high-value utilization of seafood by-products.

## CRediT authorship contribution statement

**Haoyu Liu:** Writing – original draft, Formal analysis, Data curation. **Jiaxin Chen:** Investigation, Formal analysis, Data curation. **Ruoyu Xie:** Visualization, Software. **Jun Hu:** Resources, Methodology. **Hongying Du:** Resources, Conceptualization. **Xinyan Peng:** Supervision, Resources. **Zhengshun Wen:** Resources, Conceptualization. **Xiaolong Ji:** Supervision, Methodology. **Wenxuan Chen:** Supervision, Project administration, Funding acquisition. **Jin Zhang:** Writing – review & editing, Writing – original draft, Supervision, Project administration, Funding acquisition, Conceptualization.

## Declaration of competing interest

The authors declare that they have no known competing financial interests or personal relationships that could have appeared to influence the work reported in this paper.
